# Resolving the ‘nitrogen paradox’ of *arbuscular mycorrhizas*: fertilization with organic matter brings considerable benefits for plant nutrition and growth

**DOI:** 10.1111/pce.12667

**Published:** 2016-02-10

**Authors:** Tom J. Thirkell, Duncan D. Cameron, Angela Hodge

**Affiliations:** ^1^ Department of BiologyUniversity of YorkWentworth WayYorkYO10 5DDUK; ^2^ Department of Animal and Plant SciencesUniversity of SheffieldWestern BankSheffieldS10 2TNUK

**Keywords:** arbuscular mycorrhiza, growth, nitrogen, nitrogen paradox, organic matter, symbiosis

## Abstract

Arbuscular mycorrhizal fungi (AMF) can transfer nitrogen (N) to host plants, but the ecological relevance is debated, as total plant N and biomass do not generally increase. The extent to which the symbiosis is mutually beneficial is thought to rely on the stoichiometry of N, phosphorus (P) and carbon (C) availability. While inorganic N fertilization has been shown to elicit strong mutualism, characterized by improved plant and fungal growth and mineral nutrition, similar responses following organic N addition are lacking. Using a compartmented microcosm experiment, we determined the significance to a mycorrhizal plant of placing a ^15^N‐labelled, nitrogen‐rich patch of organic matter in a compartment to which only AMF hyphae had access. Control microcosms denied AMF hyphal access to the patch compartment. When permitted access to the patch compartment, the fungus proliferated extensively in the patch and transferred substantial quantities of N to the plant. Moreover, our data demonstrate that allowing hyphal access to an organic matter patch enhanced total plant N and P contents, with a simultaneous and substantial increase in plant biomass. Furthermore, we demonstrate that organic matter fertilization of arbuscular mycorrhizal plants can foster a mutually beneficial symbiosis based on nitrogen transfer, a phenomenon previously thought irrelevant.

## Abstract 2

When granted access to an organic matter patch, arbuscular mycorrhizal fungi were found to obtain significant amounts of nitrogen (N) from the patch and transfer a substantial amount to their partner plant. The patch‐derived N contributed significantly to, and increased, total plant N. Total plant phosphorus (P) increased also, as did total plant mass. This study is the first to demonstrate simultaneous increases in N, P and biomass of plants with arbuscular mycorrhizal hyphal access to a patch of organic matter.

## Introduction

The arbuscular mycorrhizal (AM) association is the most common type of mycorrhizal symbiosis and forms between c. two‐thirds of all land plant species and soil fungi in the phylum Glomeromycota. The fungus receives photosynthetically fixed carbon (C) while, in return, the fungus confers a number of benefits to its associated host plant, the most well‐established being that of increased acquisition of phosphorus (P) (Smith & Read 2008). More recently, however, there has been renewed interest in the ability of arbuscular mycorrhizal fungi (AMF) to supply nitrogen (N) to their associated host plant and the implications this may have for N cycling (reviewed by Hodge & Storer 2015).

While it has been shown that AMF can transfer N to their associated host (Ames *et al*. 1983; Hodge *et al.* 2001; Barrett *et al*. 2011) significant doubts remain as to the ecological relevance of such a AMF–N uptake pathway (see Read 1991; Smith & Smith 2011). In particular, regarding the exact mechanism of N transfer and, more importantly, the amounts of N transferred via the AMF compared to the N requirements of the plant (Smith & Smith 2011). Although results from root organ culture studies suggest values of up to 50% of root N may be acquired via the AMF route (Govindarajulu *et al*. 2005), ideal as these systems are for unpicking mechanisms involved in nutrient exchange, it may be unwise to infer much about whole plant nutrient dynamics. Source–sink relationships, for example, are undoubtedly unrealistic given the growth conditions employed (Smith & Smith 2015). More realistic experiments, using whole plants and adding N as organic matter patches, have shown that AMF contribution to plant N uptake can be as high as 15–20% (Leigh *et al*. 2009; Barrett *et al*. 2014). Although this may suggest a significant nutritional contribution to the plant, the total plant N content (Hodge *et al.* 2000a, Hodge 2001, Leigh *et al*. 2009) and plant biomass (Hodge *et al*. 2001; Herman *et al*. 2012) are usually unaffected. In some cases, the plant may even suffer a reduction in biomass (Reynolds *et al*. 2005), implying providing N fertilization to N‐limited symbioses may be deleterious.

Johnson (2010) proposed the ‘trade balance model’ to explain the apparent ‘nitrogen paradox’, where nitrogen fertilization of AM plants causes apparent mycorrhizal parasitism of partner plants. Fundamentally, the model states that the relative supply of C from the plant and availability of N and P in the soil determines the extent to which the AM‐route for N uptake is mutually beneficial. The model suggests that fertilization with N is only beneficial if the plant is limited by P and will therefore benefit from providing C to the roots and mycorrhizal fungi.

Positive growth responses to N fertilization have been shown in plants receiving inorganic N inputs (Johnson *et al*. 2014), but corroborating evidence for an AM‐mediated plant growth response after being fertilized with organic N is lacking. Addressing this knowledge gap is now pressing, given the ecological role of AMs in nitrogen cycling (Hodge, 2014; Hodge & Storer 2015) and the nature of soil N. Most rhizosphere N is bound in complex, organic material (Bremner 1949, Stevenson, 1994) and only a small, ephemeral pool of inorganic nitrogen exists at any given time, and inorganic N turnover in soil is rapid (Jackson *et al*. 1988). The integrity of the trade balance model in systems fertilized with organic N is thus far untested.

Organic N fertilization is receiving increased attention in both research and agriculture with the adoption of more sustainable agricultural practices not only in Europe but across the world (Matson *et al*. 1997). Inorganic N fertilization may reduce mycorrhizal inoculum potential of agricultural soil (Liu *et al*., 2012); increase pathogen severity (Matson *et al*. 1997) and boost greenhouse gas fluxes from agricultural soils (McSwiney & Robertson 2005). Combining organic N fertilization and the mycorrhizal symbioses may be useful in negating some of these problems and increasing assimilation of fertilizer N into plants which is currently limited to around 40–60% in crop plants to which inorganic N is applied (Huber & Watson 1974, Paustian *et al*. 1995).

Given the abundance of AMFs in temperate soils and the range of plant species they may colonize (Smith & Read 2008), it is surprising that we do not comprehensively know which forms of soil nitrogen can be utilized by the fungus. It is well established that AMF acquire inorganic N as NH_4_^+^ and NO_3_^−^ (Govindarajulu *et al*. 2005; Leigh *et al*. 2011; Johnson *et al*. 2014), which represent the most abundant available inorganic sources in the hyphosphere (Tinker & Nye 2000). How commonly AMF utilize organic N directly is less well known. Experiments have shown that AMF may be capable of direct glycine uptake (Hawkins *et al*. 2000; but see Hodge 2001; Whiteside *et al*. 2012), and Cappellazzo *et al.* (2008) identified an amino acid permease in *Glomus mosseae*, a mechanism by which an AMF may acquire organic N directly from soil substrates. Similarly, Belmondo *et al*. (2014) show evidence for potential uptake of organic N by a dipeptide transporter in the extraradical mycelium of *Rhiozphagus irregularis*. However, AMF seem not to acquire organic N exclusively or indeed preferentially, as ^13^C enrichment is usually not detected in AM hyphae or plant tissue following hyphal access to ^13^C:^15^N dual labelled organic matter (Hodge & Fitter 2010, Nuccio *et al*. 2013).

By their very nature, complex organic matter patches contain a mixture of organic and inorganic sources of N. Both inorganic N and the simplest organic N components are likely to be relatively labile and more easily mobile in the soil than larger organic, nitrogenous constituents (Nemeth *et al*. 1987). In microcosm experiments with separate root and hyphal compartments, the potential for the N‐rich, labile fraction from organic matter patches to leach from one compartment to another presents uncertainty. This is compounded as there remains in the literature a lack of patch analysis to show the relative composition of the patch (organic versus inorganic N).

In this experiment a patch of ^15^N labelled algal material was used in order that the amount of N acquired by the plant from the patch could be measured. Algae was used owing to its low C:N ratio of 7:1, representing a rich N source. Compartmented microcosms were employed to investigate the effect of a discrete zone or ‘patch’ of N‐rich organic matter to a sand and clay growth medium of low‐N and low‐P availability. Mycorrhizal plants were contained in one compartment while AMF hyphae were permitted access to a second compartment containing the algal patch. Control microcosms in which the AMF could not access the patch were included in order that N movement via mass flow and diffusion could be determined.

## Materials and Methods

### Microcosm design

**Figure 1:**
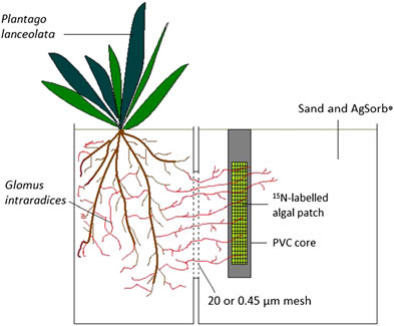
Diagram of the microcosm design. An individual *Plantago lanceolata* plant, colonized by *Glomus intraradices* was contained within one compartment of the microcosm, and a ^15^N‐labelled organic matter patch was placed into an adjoining patch compartment. Patches were contained within a PVC core, and retained within mesh sides which allow arbuscular mycorrhizal fungal (AMF) hyphal entry. Half of the microcosm units contained a 0.45 *μ*m mesh rather than a 20 *μ*m mesh, to prevent the roots and the AMF hyphae crossing from the plant compartment to the patch compartment. This allowed for any mass flow and diffusion of ^15^N from the patch across the barrier to be accounted for, rather than genuine transfer via the AMF hyphae.

Microcosm units were constructed by fastening together two polypropylene boxes, adapted from Hodge & Fitter (2010). The plant compartment measured 7 × 14 × 16 cm, and the patch compartment 14 × 14 × 16 cm. A window cut in the abutting sides of the boxes created an aperture (4 × 6 cm) that was covered with a double‐ply mesh barrier. The ‘Arbuscular Mycorrhizal Access’ (AMA) units used a 20 *μ*m mesh (John Stanier and Co., Whitefield, Manchester, UK) barrier, which prevented root access but allowed AMF hyphal access. The ‘No Arbuscular Mycorrhizal Access’ (NAMA) units used a 0.45 *μ*m mesh (Anachem, Bedfordshire, UK) that prevented the access of both roots and AMF hyphae to the patch compartment. This 0.45 *μ*m mesh barrier does not retard the diffusion of solutes from the patch compartment to the plant compartment, but the AMF mycelium cannot encounter the organic matter patch directly in NAMA microcosms. Into the bottom of each compartment, four holes were drilled and covered with 20 *μ*m mesh to permit drainage. Both compartments of the microcosms were filled with a 1:1 (*v/v*) mix of silica sand and AgSorb® [a calcinated, attapulgite clay soil conditioner, Oil‐Dri, Cambridgeshire, UK (formerly TerraGreen®; see Hodge *et al*. 2000a)]. Both sand and AgSorb® were washed 3 times in de‐ionized water prior to mixing, in order to minimize mobile mineral ions in the growth medium. Within the patch compartment of the microcosm units, the organic matter was contained inside a PVC pipe of diameter 2 cm and height 7 cm, which has two windows cut into the sides, creating two apertures each with dimensions 4 cm (H) × 1 cm (W). These apertures were covered in the same 20 *μ*m mesh as detailed above (and see Field *et al*. 2012). Such a setup ensures a uniform patch size across all microcosms, permits AMF hyphal access and allows easy placement of the organic matter patch (Fig. 1).

*Plantago lanceolata* L. was selected as the host plant owing to its ability to become highly colonized by AMF. Seeds of *P. lanceolata* (Emorsgate Wild seeds, Nottingham, UK) were surface sterilized in a 5% (*w/v*) calcium hypochlorite solution, after which they were germinated on filter paper in a sterile Petri dish. At week 0, 2‐week‐old seedlings were transferred into each plant compartment, 4 cm from the mesh aperture (three seedlings were planted into each microcosm, subsequently thinned to a single seedling at week 2). The plant compartment was watered daily, as required, with de‐ionized water.

Except for an aperture through which the plants grew, microcosms were enveloped in aluminium foil to reduce the influx of contaminating organisms. The microcosms were planted on 30 August 2013, and maintained in a heated, lit glasshouse and re‐randomized weekly to avoid environmental artefacts. From planting to harvesting, the experiment ran for 23 weeks.

### AMF inoculum

Into the plant compartment, 50 g of *Glomus intraradices* inoculum comprising macerated *P. lanceolata* and *Trifolium repens* L roots inoculated with *G. intraradices* (isolate BB‐E; Biorhize, Dijon, France) and growth medium (sand and AgSorb® mix described above) was added. The mycorrhizal inoculum was 9 months old when added to microcosms. While we acknowledge that changes in the nomenclature of AMF species have been recommended (see Redecker *et al*. 2013), here, we retain the previous name ‘*G. intraradices*’, given that the exact phylogenetic position of this particular isolate is uncertain.

### Nutrient addition

Each week, the plant compartment of each microcosm received 50 mL of a low‐N and low‐P nutrient solution (as Leigh *et al*. 2009) containing 2.5 mmol L^−1^ N as NH_4_NO_3_ and 0.034 mmol L^−1^ P as NaH_2_PO_4_. The pH of this nutrient solution was adjusted to 7.0 with KOH. The plant compartment also received 0.25 g L^−1^ bone meal (Vitax, Leicestershire, UK), a complex N and P source which encourages AM establishment (Hodge & Fitter 2010). Bone meal was added only once, at the start of the experiment. Over the course of the experiment, the nutrient solution added to the plant compartment provided 112 mg N and 2.16 mg P. The bone meal provided 23 mg N and 58 mg P. The patch compartment received no further nutrient additions after the patches had been placed.

### Patch material

After 16 weeks of plant growth in the microcosms, patches of organic litter were added to the patch compartment, 6 cm away from the mesh aperture between compartments. Each patch contained 0.075 g of 98 Atom% ^15^N‐labelled algae (obtained from Sigma‐Aldrich, St Louis, MO, USA) in a matrix of 0.8 g homogenized algal matter (*Chlorella variabilis* – PinkSun Essentials and Organics, Clayton, Yorkshire, UK). The patch contained 59 mg N (8.85 mg of which was ^15^N), 26 mg P, 413 mg C and the C:N ratio of the organic matter patch was 7:1.

The organic patch was mixed into 20 g of the silica sand:AgSorb® mix, which was then placed into the PVC pipe, filling the bottom 5 cm. The remaining 2 cm of PVC core was filled with the sand: Agsorb® growth medium only. The PVC pipe was placed into the patch compartment to a depth of 7 cm, such that the top of the core was flush with the level of the growth medium in which it sat.

Labile nitrogen as ammonium or nitrate in the algal patch was quantified by spectrophotometer (CECIL 100 spectrophotometer, Spectronic Analytical Instruments, Leeds, UK) and calculation from standard curve, created using standards containing 10 mg N L^−1^ made from NH_4_Cl and KNO_3_. Briefly, 0.2 g algal material was mixed with 10 mL de‐ionized water and incubated for 60 min at 50 °C. This preparation was then centrifuged at 5000 *g* for 15 min, after which the supernatant was decanted. Labile nitrate was measured as detailed in Cataldo *et al*. (1975). Briefly, a 0.2 mL aliquot of supernatant was placed in a 50 mL Erlenmeyer flask, to which 0.8 mL 5% (*w/v*) salicylic acid in >96% (*v/v*) H_2_SO_4_ was added. After cooling to 20 °C the flask received 19 mL of 2 M NaOH to raise the pH above 12. Absorbance was measured at 410 nm, after samples had cooled to 20 °C. Labile ammonium quantification required the use of the solutions ‘A’ and ‘B’, with details of preparation given below. A 0.05 mL aliquot of supernatant was mixed with 1 mL ‘solution A’, 0.25 mL ‘solution B’ and 2.5 mL de‐ionized H_2_O. Both solutions ‘A’ and ‘B’ were prepared using de‐ionized water. Solution A contained 20 g trisodium citrate dihydrate, 17 g salicylic acid, 5 g NaOH and 0.2 g sodium nitroprusside, made up to 500 mL. Solution B, also made up to 500 mL, contained 5 g NaOH and 0.4 g dichlorosyonurate. Absorbance was measured by spectrophotometer at 650 nm.

### Harvest

At 23 weeks after planting, the systems were destructively harvested. The *P. lanceolata* was separated into shoots and roots, and dried at 80 °C for 48 h. A subsample of the extracted roots was retained to assess root length colonization by the AMF. After drying, root and shoot material was ground and homogenized in a ball mill (Retsch MM400, Retsch GmbH, Haan, Germany), for analysis by Isotope Ratio Mass Spectrometry (PDZ 2020, Sercon Ltd, Crewe, UK).

Phosphorus content was measured using X‐ray fluorescence spectrometry (XRF). Briefly, dried plant material was milled and homogenized as described above, before being pressed into a pellet and analysed with a portable X‐ray fluorescence spectrometer (as Reidinger *et al*. 2012).

Mycorrhizal roots were stained using the method of Kormanik & McGraw (1982). Roots were cleared in 10% (*w/v*) KOH, acidified in 1% (*v/v*) HCl, stained with acid fuchsin and then stored in destain solution (lactic acid, glycerol, distilled H_2_O 10:1:1). All procedures were incubated at 20 °C, as per the ‘no heating’ variation of the method, detailed by Kormanik & McGraw (1982).

To quantify AMF extraradical mycelium, 5 g samples of growth medium were taken from the plant compartment, from within the PVC pipe containing the organic matter patch, and from the ‘bulk’ growth medium (i.e. within the patch compartment but outside the PVC core). Hyphal extraction was carried out by the modified membrane filter technique of Staddon *et al* (1999), and hyphal length assessed using the gridline intercept method from which hyphal length densities were then calculated (Hodge 2003).

### Statistical analysis

All data were analysed using SPSS 21 (IBM SPSS Inc. Armonk, NY, USA), utilizing Levene's test for equality of variance. Data for HLD in bulk versus plant compartments were analysed using Paired‐Sample T Tests, while all other data were analysed using Independent‐Samples T Test. Data were transformed to satisfy Kolmogorov–Smirnov and Shapiro–Wilk tests of normality. Percentage data were square root‐arcsine transformed before analysis.

## Results

**Figure 2:**
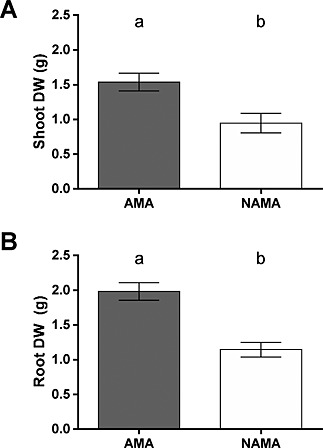
Dry weight (DW) (g) of *Plantago lanceolata* colonized by *Glomus intraradices* in Arbuscular Mycorrhizal fungal Access (AMA) and No Arbuscular Mycorrhizal fungal Access (NAMA) microcosms. a) Allowing arbuscular mycorrhizal fungal hyphal access to the patch compartment resulted in a significant increase in shoot DW (*P* = 0.001). b) Plant root DW was significantly greater for AMA plants than for NAMA (*P* < 0.001). Data shown are means ± SE, *n* = 19. Different letters above bars indicate significantly different means (*P* < 0.05).

Total plant dry weight increased substantially when AMF hyphae were allowed access to the patch compartment (3.44 ± 0.21 g with access versus 2.09 ± 0.23 g without access, *T*_1,37_ = 4.33, *P* < 0.001). This increase in plant dry weight was driven by an increase in both the shoot and root mass, which increased by 62% and 73%, respectively, compared with those plants whose AMF partner was not permitted access to the organic patch (Fig. 2). There was, however, no significant difference in the root weight ratio (RWR; ratio of root dry weight to total plant dry weight) between any treatments, suggesting that allocation of biomass between roots and shoots did not change as a result of the plants' AMF partner having access to the organic material substrate.

**Figure 3:**
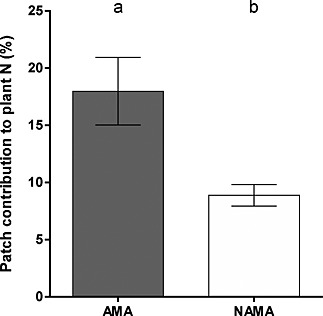
The proportion of plant N that was derived from the organic patch was greater in plants with Arbuscular Mycorrhizal fungal Access (AMA) to the patch than in those plants with No Arbuscular Mycorrhizal fungal Access (NAMA) to the patch (*P* = 0.001). Data shown are means ± SE, *n* = 19. Different letters above bars indicated significantly different means (*P* < 0.05).

Allowing AMF hyphal access to the patch greatly increased the plant uptake of ^15^N, measured both in the shoot and the root of the partner plants (Table 1). In total, plants with AMF access to the patch contained 1.10 ± 0.25 mg ^15^N compared with 0.34 ± 0.12 mg (*T*_1,37_ = 4.91, *P* < 0.001) in the plants whose AMF partner was denied access to the patch. The presence of ^15^N in the No AMF Access microcosm plants (Table 1) is ascribed to the mass flow and diffusion of ^15^N‐containing molecules through the 0.45 *μ*m mesh from the patch to the plant compartment. Such a difference in plant ^15^N content between AMF Access and No AMF Access microcosms highlights the important role AMF can play in nutrient acquisition from nutrient‐rich areas placed at significant distances beyond the rhizosphere. Similarly, the contribution made by patch N to overall plant N was greatly increased when plants had AMF access to the patch (Fig. 3): 18 ± 3%, compared to 9 ± 1% (*T*_1,37_ = 3.57, *P* = 0.001). *P. lanceolata* benefitted greatly from this AMF contribution acquired from the patch as demonstrated by the 68% increase in shoot N content when AMF had access to the patch compartment, corroborated by an 80% increase in root N content (Table 1). Thus total N in the whole plant was increased 76%. Although total plant N content increased, plant N concentration was not significantly different between the two AMF access treatments. The proportion of the patch N acquired by the plant increased from 4% to 12% when AMF were permitted access to the patch compartment (*T*_1,37_ = 4.98, *P* < 0.001), suggesting that the AMF were adept at exploiting a newly available patch of organic matter and transferring the N acquired to their plant partner. Allowing AMF hyphal access to the patch resulted in 4.69 mg extra N in the roots and 4.61 mg in the shoots which greatly outweighs the 0.001124 mg of ammonium‐N and 0.0003976 mg nitrate‐N extractable from the patch.

**Table 1:** The consequence of the arbuscular mycorrhizal fungus (AMF) *Glomus intraradices* hyphae being permitted access to the patch on *Plantago lanceolata* nutrient acquisition. Data presented are values per plant, for microcosms allowing AMF Access (AMA) versus No AMF Access to the patch (NAMA), measured 16 weeks after patch addition. Allowing AMF access to the organic matter patch allowed the plant greater uptake of ^15^N, phosphorus (P) and nitrogen (N). Data were analysed by Independent‐samples T Test, and data shown are means (*n* = 19 for N measurements; *n* = 17 for P measurements) ± S.E

	Shoot ^15^N content (mg)		Root ^15^N content (mg)		Shoot N content (mg)		Root N content (mg)		Shoot P content (mg)		Root P content (mg)	
AMA	0.57 ± 0.15		0.53 ± 0.11		11.43 ± 1.11		10.53 ± 0.69		9.92 ± 0.93		3.48 ± 0.33	
NAMA	0.20 ± 0.08		0.14 ± 0.04		6.82 ± 1.46		5.84 ± 0.69		5.12 ± 0.59		1.48 ± 0.14	
Test statistics	*T*_1,37_	*P*	*T*_1,37_	*P*	*T*_1,37_	*P*	*T*_1,37_	*P*	*T*_1,34_	*P*	*T*_1,34_	*P*
	3.99	0.001	5.96	< 0.001	3.77	< 0.001	5.02	< 0.001	4.98	< 0.001	5.83	< 0.001

Root P concentration increased by 28% when AMF had access to the patch (*T*_1,34_ = 3.31, *P* = 0.002), but the shoot P concentration was not affected by allowing AMF access to the patch. The increase in root P concentration was not substantial enough to change the total plant P concentration (*T*_1,34_ = 0.16, *P* = 0.88), but combined with an increased root mass, root P content increased by 135%. Similarly, despite no increase in P concentration, shoot P content was 94% greater in AMF access plants than in no AMF access plants (Table 1). Plants with AMF access to the patch had marginally higher N:P ratios (total plant N content/total plant P content) than plants with no patch access although this was only weakly significant (*T*_1,34_ = 1.98, P = 0.060). Mean AMA plant N:P was 2.18 ± 0.15, compared with mean NAMA plant N:P of 1.84 ± 0.10.

**Figure 4:**
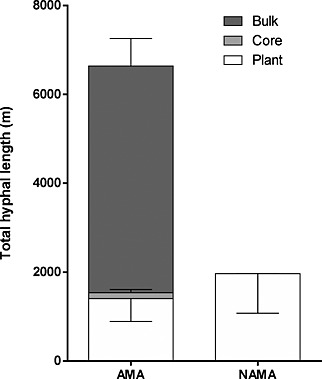
Total hyphal length, extrapolated from hyphal length density (HLD) measurements. HLD was calculated from growth medium in the PVC core, from the plant compartment and the bulk growth medium that surrounded the core. Plants with Arbuscular Mycorrhizal fungal Access (AMA) supported substantially more total hyphal length than those plants with No Arbuscular Mycorrhizal Access (NAMA), despite plant compartment hyphal length being higher in NAMA microcosms than AMA. Total hyphal length in the bulk growth medium surrounding the core was higher than in the plant compartment when AMF hyphae were permitted access to the compartment containing the patch (*T*_1, 18_ = 4.94, *P* < 0.001). (Data shown are means ± SE, *n* = 19).

Although low levels of fungal hyphae (0.01 ± 0.01 m g^−1^ DW) were found in the organic matter patches where AMF were denied access, hyphal length densities (HLD) were significantly greater (*T*_1,37_ = 18.67, *P* < 0.001) in the treatments that permitted AMF hyphal access to the organic matter patch (1.54 ± 0.19 m g^−1^ DW). Hyphal growth in the plant compartment was 21% greater when the AMF partner was denied access to the patch compartment than when access was permitted (Fig. 4). In AMA microcosms, hyphal proliferation in the bulk growth medium was significantly greater than in the plant compartments (*T*_1,18_ = 4.94, *P* < 0.001) suggesting that the C supply from the plant was limited and that the fungus was optimizing distribution of its hyphal network – into the patch compartment instead of the plant compartment. Calculating total hyphal length (by extrapolating from the HLD in compartments, assuming equal distribution of hyphae within compartments) shows that the AMA microcosms supported in excess of three times the hyphae seen in the NAMA microcosms (Fig. 4). The higher HLD in the NAMA plant compartments suggests that this was not because of reaching a maximum attainable density in this compartment, and supports the notion of limited C supply to the AMF mycelium.

^15^N content in the growth medium outside the patch did not change between treatments, even in the plant compartment, suggesting that ^15^N lost from the patch was either lost as volatile constituents to the atmosphere, or that the AMF was very successful at acquiring N from the patch. Unfortunately it was not possible to quantify the root length colonization because of disintegration of the root material during the clearing process.

## Discussion

We show for the first time that both total N content and total dry weight of plants increased as a result of allowing AMF access to an organic matter patch. Our data show that an organic N source can elicit the ‘strong mutualism’ scenario predicted by the ‘trade balance model’ of Johnson (2010), whereby both plant and fungi benefit from the addition of a rich N source in a P‐limited system. Previous work has shown mutual benefit, but only following inorganic N addition (Johnson *et al.* 2014). Here, *G. intraradices* was adept at acquiring N from patches of algal material and transferring a significant fraction of patch N to the partner plant. The quantity of nitrogen transferred, and the increased contribution made by the ERM in AMA microcosms support the argument that AMF can be a significant conduit for plant N uptake, a position not universally supported in the literature (e.g. Reynolds *et al.* 2005). Although AM uptake of N from organic matter patches has been reported (Leigh *et al.* 2009, Barrett *et al*. 2014), previous studies generally have not displayed increased total plant N, as we show, and a concurrent increase in plant biomass as found in this study is unprecedented. The increase of ^15^N in the plant shoots (Table 1) is noteworthy as it indicates genuine transfer of patch N to the plant via the AMF, whereas it cannot be determined what proportion of ^15^N in roots remains in the intraradical mycelium of the AMF. We assumed no fractionation of ^15^N and ^14^N during uptake by the fungus or transfer to the plant. Ectomycorrhizal fungi may transfer ^14^N preferentially to a partner plant, so the mycelium becomes relatively ^15^N enriched (Hobbie & Colpaert 2003). Were such a phenomenon to have taken place here, our calculations for N transfer would be underestimating the contribution made by the AMF to plant N.

The increased contribution of patch N to the plants' total N when AMF were allowed access the patch was similar to that shown by Leigh *et al.* (2009), suggesting that the AMF was at least as able to exploit algal patches as *Lolium perenne* patches, as used by Leigh *et al.* (2009). Where that study showed increased plant N concentration however, we saw increased total N content and plant mass, but no difference in N concentration between treatments. Differences in patch composition may explain different responses of the AM plant, despite using the same plant and fungal symbiont species. The low C:N ratio of our patch compared to that used by Leigh *et al.* (2009) makes our patch more N‐rich, and should therefore allow more rapid loss of the N it contained (Hodge *et al.* 2000b). Rapid efflux of N from the patch is suggested by the reasonably high level of ^15^N detected in the plant tissue from NAMA microcosms (Table 1). Movement of labile N sources by mass flow and diffusion across the 0.45 *μ*m membrane from patch to plant compartment is implicated, but higher N and P levels in AMA treatments confirm the importance of AMF mediated nutrient transfer. Although it remained inside the PVC tube, the algal powder settled and mixed with the sand and AgSorb® during the course of the experiment, and became inseparable from the latter by the time the microcosms were harvested. As such, the retrieval of the patch at the end of the experiment was not possible. This prevented patch analysis to determine the extent of decomposition.

The contribution of patch N to total plant N varies among different studies using similar experimental systems: from <7% (Hodge & Fitter 2010; Barrett *et al*. 2011; Herman *et al*. 2012) to >15% (Barrett *et al*. 2014; Leigh *et al*. 2009; this study). Some of these differences can be explained by variation among different AMF symbionts (e.g. Leigh *et al*. 2009; Barrett *et al*. 2014). However, the AMF may also benefit the plant from acquiring ‘extra’ N from sources other than the patch (Herman *et al*. 2012). Increased P uptake by AMA plants is perhaps expected, given the amount of P present in the patch and that AMs probably evolved to improve the uptake of immobile ions, such as phosphate, from soil beyond the rhizosphere (Smith & Read 2008). In this study, the N:P ratios were remarkably low, but not without precedent for forbs (Maloney & Lamberti 1995), and indicate that the plants were severely N‐limited. The increase in N:P ratio in AMA plants compared with NAMA plants suggests that the AMF reduced the extreme N limitation the plants were experiencing and in so doing facilitated growth benefits for the plant. Leigh *et al.* (2009) showed no difference in N:P between AMA and NAMA plants, suggesting that the AMF in that case did less to lift the plant from N‐limitation, and offering an explanation as to why no growth response was observed there.

Increased P content in AMA plants suggests that the patch represented a significant source of P for the fungus (see also Barrett *et al*. 2014). Cavagnaro *et al.* (2005) demonstrated that *G. intraradices* proliferated in high P patches, while reducing P uptake from low P areas. Hyphal proliferation in the high‐N, high‐P patch compartment and reduced AMF growth in the plant compartment in AMA microcosms (Fig. 4) suggests that the fungus behaved similarly here.

Reduced hyphal length in the plant compartment of AMA microcosms may suggest C limitation, as previous studies with similar experimental design (Hodge *et al*. 2001) showed increased growth of AMF in plant and patch compartments. Here, the fungus may have been unable to obtain enough C from the plant to maintain the hyphal biomass in the plant compartment when it was also supporting a mycelium in the patch compartment, and thus co‐ordinated its hyphal growth for the greatest benefit – the mineral nutrition from the patch, a phenomenon that is well documented in root allocation (Drew 1975).

Previously, experimental evidence for strong mutual benefit of AMs was obtained only by inorganic N addition to mycorrhizal plants. Our findings demonstrate that AMF can provide considerable benefit to plant N and P nutrition following the addition of organic matter, followed by substantial increases in biomass, both for plant and fungus.

## Conflict of Interest

The authors have no conflict of interest to declare.

## References

[pce12667-bib-0001] Ames R.N. , Reid C.P.P. , Porter L.K. & Cambardella C. (1983) Hyphal uptake and transport of nitrogen from 2 ^15^N‐labeled sources by *Glomus mosseae*, a vesicular arbuscular mycorrhizal fungus. New Phytologist 95, 381–396.

[pce12667-bib-0002] Barrett G. , Campbell C.D. , Fitter A.H. & Hodge A. (2011) The arbuscular mycorrhizal fungus *Glomus hoi* can capture and transfer nitrogen from organic patches to its associated host plant at low temperature. Applied Soil Ecology 48, 102–105.

[pce12667-bib-0003] Barrett G. , Campbell C.D. & Hodge A. (2014) The direct response of the external mycelium of arbuscular mycorrhizal fungi to temperature and the implications for nutrient transfer. Soil Biology & Biochemistry 78, 109–117.

[pce12667-bib-0004] Belmondo S. , Fiorilli V. , Pérez‐Tienda J. , Ferrol N. , Mermeisse R. & Lanfranco L. (2014) A dipeptide transporter from the arbuscular mycorrhizal fungus *Rhizophagus irregularis* is upregulated in the intraradical phase. Frontiers in Plant Science 5, 436. 25232358 10.3389/fpls.2014.00436 PMC4153046

[pce12667-bib-0005] Bremner J.M. (1949) Studies on soil organic matter. 1. The chemical nature of soil organic nitrogen. Journal of Agricultural Science 39, 183–193.

[pce12667-bib-0006] Cappellazzo G. , Lanfranco L. , Fitz M. , Wipf D. & Bonfante P. (2008) Characterisation of an amino acid permease from the endomycorrhizal fungus *Glomus mosseae* . Plant Physiology 147, 429–437. 18344417 10.1104/pp.108.117820 PMC2330287

[pce12667-bib-0007] Cataldo D.A. , Haroon M. , Schrader L.E. & Youngs V.L. (1975) Rapid colorimetric determination of nitrate in plant tissue by nitration of salicylic acid. Communications in Soil Science and Plant Analysis 6, 71–80.

[pce12667-bib-0008] Cavagnaro T.T. , Smith F.A. , Smith S.E. & Jakobsen I. (2005) Functional diversity in arbuscular mycorrhizas: exploitation of soil patches with different phosphate enrichment differs among fungal species. Plant, Cell and Environment 28, 642–650.

[pce12667-bib-0009] Drew M.C. (1975) Comparison of the effects of a localised supply of phosphate, nitrate, ammonium and potassium on the growth of the seminal root system, and the shoot, in barley. New Phytologist 75, 479–490.

[pce12667-bib-0010] Field K.J. , Cameron D.D. , Leake J.R. , Tille S. , Bidartondo M.I. & Beerling D.J. (2012) Contrasting arbuscular mycorrhizal responses of vascular and non‐vascular plants to a simulated Palaeozoic CO_2_ decline. Nature Communications 3, 835. 10.1038/ncomms1831 22588297

[pce12667-bib-0011] Govindarajulu M. , Pfeffer P.E. , Jin H. , Abubaker J. , Douds D.D. , Allen J.W. , … Shachar‐Hill Y. (2005) Nitrogen transfer in the arbuscular mycorrhizal symbiosis. Nature 435, 819–823. 15944705 10.1038/nature03610

[pce12667-bib-0012] Hawkins H.J. , Johansen A. & George E. (2000) Uptake and transport of organic and inorganic nitrogen by arbuscular mycorrhizal fungi. Plant and Soil 226, 275–285.

[pce12667-bib-0013] Herman D.J. , Firestone M.K. , Nuccio E. & Hodge A. (2012) Interactions between an arbuscular mycorrhizal fungus and a soil microbial community mediating litter decomposition. FEMS Microbiology Ecology 80, 236–247. 22224699 10.1111/j.1574-6941.2011.01292.x

[pce12667-bib-0014] Hobbie E.A. & Colpaert J.V. (2003) Nitrogen availability and colonization by mycorrhizal fungi correlate with nitrogen isotope patterns in plants. New Phytologist 157, 115–126. 10.1046/j.1469-8137.2003.00657.x 33873704

[pce12667-bib-0015] Hodge A. (2001) Arbuscular mycorrhizal fungi influence decomposition of, but not plant nutrient capture from, glycine patches in soil. New Phytologist 151, 725–734. 10.1046/j.0028-646x.2001.00200.x 33853263

[pce12667-bib-0016] Hodge A. (2003) Plant nitrogen capture from organic matter as affected by spatial dispersion, interspecific competition and mycorrhizal colonization. New Phytologist 157, 303–314. 10.1046/j.1469-8137.2003.00662.x 33873633

[pce12667-bib-0017] Hodge A. (2014) Interactions between arbuscular mycorrhizal fungi and organic material substrates. Advances in Applied Microbiology 89, 47–99. 25131400 10.1016/B978-0-12-800259-9.00002-0

[pce12667-bib-0018] Hodge A. & Fitter A.H. (2010) Substantial nitrogen acquisition by arbuscular mycorrhizal fungi from organic material has implications for N cycling. Proceedings of the National Academy of Sciences of the United States of America 31, 13754–13759. 20631302 10.1073/pnas.1005874107 PMC2922220

[pce12667-bib-0019] Hodge A. & Storer K. (2015) Arbuscular mycorrhiza and nitrogen: implications for individual plants through to ecosystems. Plant and Soil 386, 1–19.

[pce12667-bib-0020] Hodge A. , Robinson D. & Fitter A.H. (2000a) An arbuscular mycorrhizal inoculum enhances root proliferation in, but not nitrogen capture from, nutrient‐rich patches in soil. New Phytologist 145, 575–584. 10.1046/j.1469-8137.2000.00602.x 33862913

[pce12667-bib-0021] Hodge A. , Stewart J. , Robinson D. , Griffiths B.S. & Fitter A.H. (2000b) Competition between roots and soil micro‐organisms for nutrients from nitrogen‐rich patches of varying complexity. Journal of Ecology 88, 150–164.

[pce12667-bib-0022] Hodge A. , Campbell C.D. & Fitter A.H. (2001) An arbuscular mycorrhizal fungus accelerates decomposition and acquires nitrogen directly from organic material. Nature 413, 297–299. 11565029 10.1038/35095041

[pce12667-bib-0023] Huber D.M. & Watson R.D. (1974) Nitrogen form and plant disease. Annual Review of Phytopathology 12, 139–165. 10.1146/annurev.py.12.090174.001035 23249125

[pce12667-bib-0024] Jackson L.E. , Schimel J.P. & Firestone M.K. (1988) Short term partitioning of ammonium and nitrate between plants and microbes in an annual grassland. Soil Biology & Biochemistry 21, 409–415.

[pce12667-bib-0025] Johnson N.C. (2010) Resource stoichiometry elucidates the structure and function of arbuscular mycorrhizas across scales. New Phytologist 185, 631–647. 19968797 10.1111/j.1469-8137.2009.03110.x

[pce12667-bib-0026] Johnson N.C. , Wilson G.W.T. , Wilson J.A. , Miller R.M. & Bowker M.A. (2014) Mycorrhizal phenotypes and the Law of the Minimum. New Phytologist 205, 1473–1484. 25417818 10.1111/nph.13172

[pce12667-bib-0027] Kormanik P.P. & McGraw A.C. (1982) Quantification of vesicular‐arbuscular mycorrhizae in plant roots In Methods and Principles of Mycorrhizal Research (ed Schenk N.C.), pp. 37–46. American Phytopathological Society, St Paul, MN, USA.

[pce12667-bib-0028] Leigh J. , Hodge A. & Fitter A.H. (2009) Arbuscular mycorrhizal fungi can transfer substantial amounts of nitrogen to their host plant from organic material. New Phytologist 181, 199–207. 18811615 10.1111/j.1469-8137.2008.02630.x

[pce12667-bib-0029] Leigh J. , Fitter A.H. & Hodge A. (2011) Growth and symbiotic effectiveness of an arbuscular mycorrhizal fungus in organic matter in competition with soil bacteria. FEMS Microbiology Ecology 76, 428–438. 21303398 10.1111/j.1574-6941.2011.01066.x

[pce12667-bib-0030] Liu Y.J. , Shi G.X. , Mao L. , Cheng G. , Jiang S.J. , Ma X.J. ,…, Feng H.Y. (2012) Direct and indirect influences of 8 yr of nitrogen and phosphorus fertilization on Glomeromycota in an alpine meadow ecosystem. New Phytologist 194, 523–535. 22292929 10.1111/j.1469-8137.2012.04050.x

[pce12667-bib-0031] Maloney D.C. & Lamberti G.A. (1995) Rapid decomposition of summer‐input leaves in a Northern Michigan stream. American Midland Naturalist 1, 184–195.

[pce12667-bib-0032] Matson P.A. , Parton W.J. , Power A.G. & Swift M.J. (1997) Agricultural intensification and ecosystem properties. Science 277, 504–509. 20662149 10.1126/science.277.5325.504

[pce12667-bib-0033] McSwiney C.P. & Robertson G.P. (2005) Nonlinear response of N_2_O flux to incremental fertilizer addition in a continuous maize (*Zea mays* L.) cropping system. Global Change Biology 11, 1712–1719.

[pce12667-bib-0034] Nemeth K. , Bartels H. , Heuer C. & Maier J. (1987) Determination by means of EUF of the inorganic and organic soil‐nitrogen available to plants. Zuckerindustrie 112, 223–226.

[pce12667-bib-0035] Nuccio E.E. , Hodge A. , Pett‐Ridge J. , Herman D.J. , Weber P.K. & Firestone M.K. (2013) An arbuscular mycorrhizal fungus significantly modifies the soil bacterial community and nitrogen cycling during litter decomposition. Environmental Microbiology 15, 1870–1881. 23360621 10.1111/1462-2920.12081

[pce12667-bib-0036] Paustian K. , Robertson G.P. & Elliott E.T. (1995) Management impacts on carbon storage and gas fluxes (CO_2_, CH_4_) in mid‐latitude cropland ecosystems In Soil Management and Greenhouse Effect (eds Lal R., Kimble J., Levine E. & Stewart B.A.), pp. 69–84. CRC Press, Florida, USA.

[pce12667-bib-0037] Read D.J. (1991) Mycorrhizas in ecosystems. Experientia 47, 376–391.

[pce12667-bib-0038] Redecker D. , Schuβler A. , Stockinger H. , Stürmer S.L. , Morton J.B. & Walker C. (2013) An evidence‐based consensus for the classification of arbuscular mycorrhizal fungi (Glomeromycota). Mycorrhiza 23, 515–531. 23558516 10.1007/s00572-013-0486-y

[pce12667-bib-0039] Reidinger S. , Ramsey M.H. & Hartley S.E. (2012) Rapid and accurate analyses of silicon and phosphorus in plants using a portable X‐ray fluorescence spectrometer. New Phytologist 195, 699–706. 22671981 10.1111/j.1469-8137.2012.04179.x

[pce12667-bib-0040] Reynolds H.L. , Hartley A.E. , Vogelsang K.M. , Bever J.D. & Schultz P.A. (2005) Arbuscular mycorrhizal fungi do not enhance nitrogen acquisition and growth of old‐field perennials under low nitrogen supply in glasshouse culture. New Phytologist 167, 869–880. 16101923 10.1111/j.1469-8137.2005.01455.x

[pce12667-bib-0041] Smith S.E. & Read D.J. (2008) Mycorrhizal Symbiosis 3rd edn. Academic Press, London, UK.

[pce12667-bib-0042] Smith F.A. & Smith S.E. (2011) Mycorrhizas in plant nutrition and growth: new paradigms from cellular to ecosystem scales. Annual Review of Plant Biology 62, 227–250. 10.1146/annurev-arplant-042110-103846 21391813

[pce12667-bib-0043] Smith F.A. & Smith S.E. (2015) How harmonious are arbuscular mycorrhizal symbioses? Inconsistent concepts reflect different mindsets as well as results. New Phytologist 205, 1381–1384. 25420770 10.1111/nph.13202

[pce12667-bib-0044] Staddon P.L. , Fitter A.H. & Graves J.D. (1999) Effect of elevated atmospheric CO_2_ on mycorrhizal colonization, external mycorrhizal hyphal production and phosphorus inflow in *Plantago lanceolata* and *Trifolium repens* in association with the arbuscular mycorrhizal fungus *Glomus mosseae* . Global Change Biology 5, 347–358.

[pce12667-bib-0045] Stevenson F.J. (1994) Humus Chemistry. Genesis, Composition, Reactions 2nd edn. John Wiley & Sons, New Jersey, USA.

[pce12667-bib-0046] Tinker P.B. & Nye P.H. (2000) Solute Movement in the Rhizosphere. Oxford University Press, New York, USA.

[pce12667-bib-0047] Whiteside M.D. , Garcia M.O. & Treseder K.K. (2012) Amino acid uptake in arbuscular mycorrhizal plants. PLoS One 7, e47643. 23094070 10.1371/journal.pone.0047643 PMC3475604

